# Association between VEGF gene polymorphisms and breast cancer risk

**DOI:** 10.1016/j.bbrep.2025.102202

**Published:** 2025-08-11

**Authors:** Hala Osman, Mozan Hassan, Mohamed Alfaki, Ghada Haj-Ali

**Affiliations:** a Al Neelain Medical Research Center, Faculty of Medicine, Al Neelain University, Khartoum, Sudan; b Faculty of Medical Laboratory Science, Omdurman Ahlia University, Khartoum, Sudan; c Department of Genetics and Genomics, College of Medicine and Health Sciences, United Arab Emirates University, Al Ain, United Arab Emirates; d Faculty of Computer Science, Al Neelain University, Khartoum, Sudan

## Abstract

**Introduction:**

Breast cancer (BC) poses a significant global health challenge. In Sudan, the absence of a national cancer registry has resulted in an underestimation of BC incidence. BC is notably the most common cancer among Sudanese women, especially affecting those under 50, with many cases diagnosed at advanced stages. Angiogenesis, driven by vascular endothelial growth factor (VEGF), plays a critical role in the progression and recurrence of BC. This study examines the relationship between the VEGF (rs699947) gene polymorphism and BC among Sudanese women in Khartoum State in 2022. **Methodology**: A case-control study was conducted with 30 BC patients, and tissue samples were collected for molecular analysis. DNA was extracted and genotyped for the VEGF (rs699947) polymorphism using allele-specific PCR.

**Results:**

No statistically significant association was found between the VEGF-2578 C > A polymorphism and BC risk in our study population. Although the A allele was more prevalent in tumor tissues compared to normal tissues, with no significant correlation with tumor stage or grade. The study revealed that BC in Sudanese women often presents at younger ages and is predominantly invasive ductal carcinoma, with stage II being the most common.

**Conclusion:**

These findings emphasize the necessity for continued research to explore additional genetic factors and improve our understanding of BC and associated risks. Advancing early detection and prevention methods is vital, particularly for underrepresented populations. However, the small sample size in this study may limit the statistical power to detect significant associations, and thus, findings should be interpreted with caution.

## Highlights

• Breast cancer is the leading malignancy among Sudanese women, often diagnosed at advanced clinical stages.
• This case-control study investigated VEGF (rs699947) polymorphism in 30 breast cancer patients in Khartoum, Sudan.
• The heterozygous TA genotype was most common; the A allele was more frequent in tumor tissue vs adjacent normal tissue.
• Invasive ductal carcinoma was the predominant histological type, with stage II as the most frequently observed stage.
• No significant link was found in this cohort, highlighting the need for broader genetic research in diverse populations.

## 1 Introduction

Breast cancer (BC) is one of the most prevalent and invasive cancers worldwide, accounting for 670,000 deaths globally in 2022, with an incidence rate of 2.3 million cases. This number has been increasing annually worldwide, according to the World Health Organization (WHO) [1]. Socioeconomic factors can significantly influence BC outcomes. In low- and middle-income countries, survival rates are often lower, due to limited access to preventive screening and early intervention, as well as inadequate treatment and dilapidated health systems [2]. In Sudan, the true incidence of BC is unknown due to the lack of a national cancer registry. Between 1959 and 2007, BC made up 16 % of all reported malignancies in Sudan [3]. Hospital-based registries showed that BC is the most common cancer among Sudanese women, with the incidence in Khartoum state being 25.1 per 100,000 between 2009 and 2010 [4]. Additionally, about 70 % of the diagnosed cases involved women under 50 years old, and around 80 % were diagnosed at an advanced stage [5]. Genetic polymorphisms in several genes have been linked to BC, with the well-known BRCA1/2 gene showing variable associations across different ethnic groups. While some populations have reported a significant link between BRCA1/2 and BC risk, others have found no such association [6,7].

Angiogenesis plays a critical role in tumor progression, considered a hallmark of cancer, and a key driver of cancer recurrence [8]. Among the key regulators of angiogenesis is vascular endothelial growth factor (VEGF), a potent pro-angiogenic factor that promotes endothelial cell proliferation, survival, and migration. These processes contribute to both physiological and pathological angiogenesis [9]. The VEGF gene, located at 6p21.3 and consists of eight exons and seven introns and is highly polymorphic, with several variants identified in the promoter, 5′-untranslated region (5′-UTR), and 3′-untranslated region (3′-UTR) [10].

Functional genetic polymorphisms affecting VEGF gene expression can have a significant impact on BC development [11]. In BC, high microvascular density is linked to invasive carcinoma, increased metastasis, and shorter overall survival. VEGF overexpression in BC cells, often preceding invasion, correlates with advanced disease stages and poorer outcomes [12]. Furthermore, an inverse relationship exists between VEGF expression and overall survival in both node-positive and node-negative BC cases [13]. A study reported a significant association between rs699947 (−2578C > A) SNP polymorphism of the VEGF gene and BC in Saudi women [14]. Another study on Saudi women found that the homozygous variant (AA) of the VEGF gene polymorphism (−2578C > A, rs699947) is a genetic risk factor for BC. It is associated with older age (>40) and higher tumor grade (II or III) and is suggested to activate gene expression [15]. Additionally, Rezaei et al. showed that the VEGF rs699947 variant increased the risk of BC in Iranian women [16]. Moreover, a study in Iraqi females revealed that the VEGF rs699947 variant (AA) significantly increased the risk of BC [17]. On the contrary, a study by Sambyal et al. demonstrated that VEGF polymorphisms (−2578C/A, −2549I/D, −460T/C, +405C/G, -7C/T, and +936C/T) were significantly associated with reduced risk of BC [8]. Furthermore, SNPs of VEGF [−1154A/G (rs1570360), −2578C/A (rs699947), and −460T/C (rs833061)] are suggested to have a protective role against BC and are not linked to tumor aggressiveness in Moroccan females [18].

Early diagnosis of BC is crucial for reducing morbidity and mortality. Recent developments in artificial intelligence have demonstrated significant potential in revolutionizing BC diagnosis, risk assessment, and treatment strategies by incorporating multi-modal clinical and genomic information [19,20]. The actual incidence of BC in Sudan is unknown due to the absence of a national cancer registry. Previous studies worldwide have shown conflicting results; some have linked the VEGF (rs699947) gene polymorphism to BC, while others have not. Investigating the VEGF (rs699947) gene polymorphism in Sudanese women could enhance understanding of BC development, help predict pre-symptomatic disease, and assess individual susceptibility to BC and its complications. To our knowledge, no study on VEGF gene polymorphism in BC has been conducted in Sudan to date. The objective of this case-control study was to investigate the association between the frequency of the VEGF (rs699947) gene polymorphism and BC among Sudanese women in Khartoum State in 2022. Additionally, the study aims to compare the different genotypes with tumor stages and grades, as well as the distribution of genotype frequency between BC tissues and adjacent normal tissues taken from the same patient.

## 2 Materials and methods

### 2.1 Study Population

A descriptive-analytical case-control study was conducted to investigate the association between VEGF gene polymorphism and BC in Sudanese female patients. Samples were collected from AL-Sheriff Hospital in Khartoum State between April and August 2022. The study received ethical approval from the Khartoum Ministry of Health, Research Ethics Committee, under the reference number KMOH-REC-060,9–2022, and the hospital administration in September 2022. Medication history was recorded using a questionnaire, and informed consent was obtained from all participants.

Tissue samples were collected from 30 patients diagnosed with BC, with two samples taken from each patient (one from the tumor tissue and the other from adjacent normal tissue) [21]. These samples were transported to a molecular lab and preserved at −20 °C. Demographic and clinical data were gathered through questionnaires and patient communication.

## 3 Molecular analysis

### 3.1 DNA extraction

DNA extraction was performed using the guanidine chloride method, as modified by Tang et al. [22]. Briefly, 5 mm of tissue samples were sliced and placed in 15 ml Falcon tubes. The samples were treated with lysis buffer, guanidine chloride, ammonium acetate, and proteinase K, and incubated overnight at 37 °C. Chloroform was added, and the mixture was vortexed, left to stand for 5 min, and centrifuged at 4000 rpm for 10 min. The DNA-containing upper layer was transferred to a new tube, mixed with ethanol, and refrigerated overnight. After centrifugation, the supernatant was discarded, and the DNA pellet was washed twice with 70 % ethanol. The pellet was dried for 1–2 h, then resuspended in 50 μL double-ionized water, and stored at −20 °C for future molecular studies [22].

### 3.2 Quality check and quantification of DNA

To assess DNA quality, a 1 % agarose gel was prepared by dissolving 1 g of agarose in 100 ml of 1X Tris-Borate-EDTA (TBE) buffer and heating in a microwave. After cooling to 55 °C, 3 μL of ethidium bromide was added to visualize the DNA. The gel was poured into an electrophoresis tray, allowed to solidify, and then loaded with 3 μL of the extracted DNA mixed with bromophenol blue dye. The gel was run at 120V and 40A for about 20 min, and samples showing sharp, bright bands were considered to have good quality. DNA quantity was measured using a nanodrop device by loading 2 μL of the DNA sample and measuring the absorbance at 260 and 280 nm.

## 4 Genotyping detection

### 4.1 Polymerase chain reaction (PCR)

Molecular genotyping was performed using allele-specific PCR to amplify the VEGF (RS699947) gene, using (Maxime PCR PreMix Kit, i-tag), Intron Biotechnology. The reaction mixture included master mix, allele-specific forward primers, a common reverse primer, DNA, and distilled water. Two separate forward primers were used: one specific for the wild-type allele (T) with the sequence 5′-TCAGTCTGATTATCCACCCACAGATCT-3′, and one for the mutant allele (A) with the sequence 5′-TCAGTCTGATTATCCACCCACAGATCA-3’. A common reverse primer was used in both reactions: 5′-CTAGTGCACGAATGGAAAGG-3’. The DNA was amplified using a thermal cycler with a specific program: initial denaturation at 94–95 °C for 5 min, 35 cycles of denaturation at 94 °C for 30 s, annealing at 60 °C for 60 s, extension at 72 °C for 10 min, and a final extension at 72 °C for 5 min. The PCR product, expected to be 295 bp in length, was identified using 2 % agarose gel electrophoresis [23]. Since all amplicons are approximately 295–300 bp in size, genotype determination was based on allele-specific primer amplification patterns, not on band size differences. A band only with the wild-type primer indicated TT genotype; only with the mutant primer indicated AA; bands with both indicated TA genotype.

### 4.2 Validation of allele-specific PCR

To ensure the reliability and specificity of allele-specific PCR, normal tissue adjacent to the tumor (safety margin) was used as an internal biological control for patients’ genotypes. A no-template control (NTC) was included in each run to rule out contamination and non-specific amplification. Additionally, all PCR reactions were performed in triplicate to confirm the reproducibility of amplification patterns.

### 4.3 Statistical analysis

The results were analyzed using SPSS version 26. Descriptive statistics were used to analyze demographic data. The association between genotypes in tumor and normal tissues and the histopathological data of BC was compared using the Chi-square test. Hardy-Weinberg Equilibrium (HWE) analysis was performed for genotype frequencies in the adjacent normal tissues using R Software with the HardyWeinberg package. Tumor tissue was excluded from HWE analysis due to potential somatic mutations and clonal evolution, which violate HWE assumptions.

## 5 Results

### 5.1 Demographic and pathological data

**Fig. 1:**
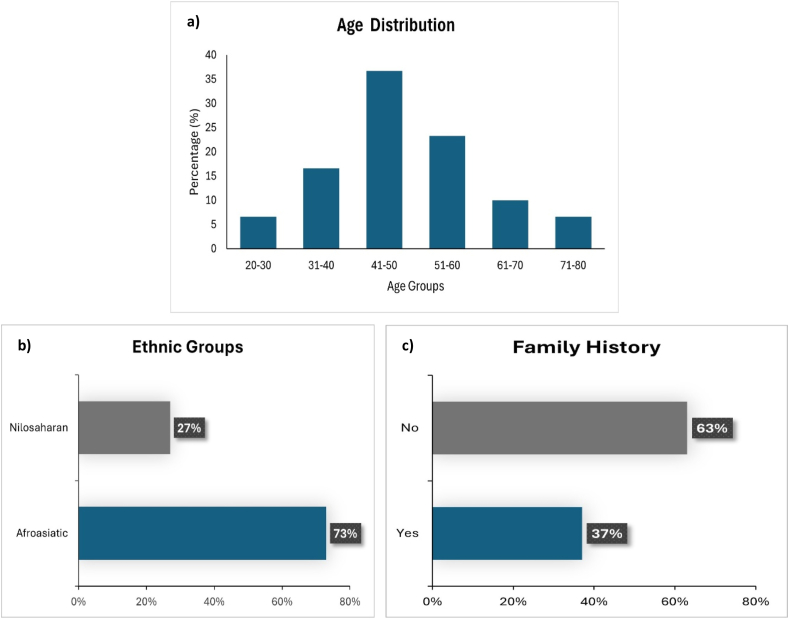
Demographic Characteristics of the Study Population. a) Age distribution of patients in percentage across defined age groups. b) Ethnic distribution showing the proportion of Afroasiatic and Nilosaharan groups. c) Family history of disease among patients, with 37 % reporting a positive family history.

The analysis of demographic data in this study included age, ethnic background, family history, and histopathological characteristics of the disease. The ages of the patients ranged from 20 to 80 years, with the highest percentage (36.7 %) falling within the 41–50 age group, and the mean age was 45 years. The subsequent age groups were 51–60, 31–40, 61–70, 71–80, and 20–30 years (Fig. 1a). The study was divided into two ethnic groups: Afro-Asiatic and Nilo-Saharan. The majority of patient subjects were Afro-asiatic, which represented 73 % followed by Nilo-Saharan represented 27 % (Fig. 1b). Regarding family history, 63 % of the patients had no family history of BC, while 37 % reported a positive family history of the disease (Fig. 1c).

**Fig. 2:**
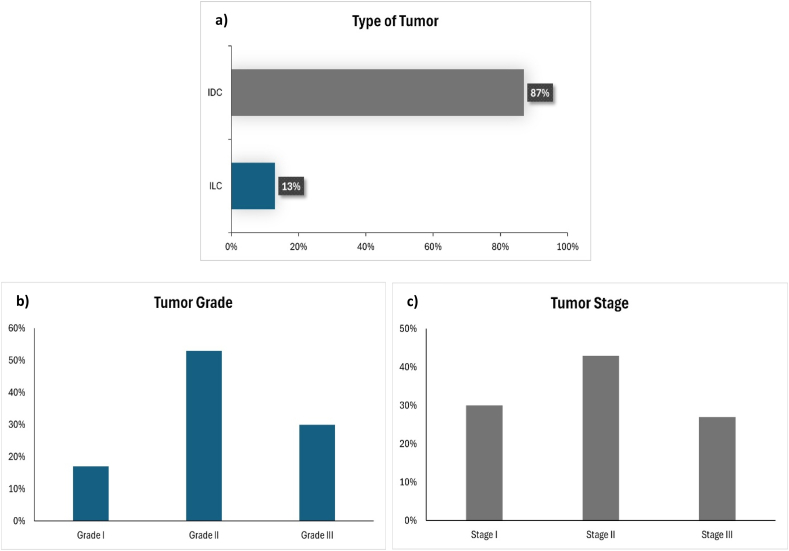
**Histopathological characteristics of breast tumors.** a) Tumor type distribution: invasive ductal carcinoma (IDC) and invasive lobular carcinoma (ILC). b) Tumor grades: most tumors were Grade II. c) Tumor stages, with Stage II being most prevalent.

Histopathological analysis showed that invasive ductal carcinoma (IDC) was the most prevalent tumor type, accounting for 87 % of the cases, while invasive lobular carcinoma (ILC) represented 13 % of the cases among Sudanese female patients (Fig. 2a). In terms of tumor grade, the majority of patients (53 %) had grade II tumors, followed by grade III (30 %) and grade I (17 %) tumors (Fig. 2b). Tumor staging revealed that stage II was the most common, observed in 43 % of patients, followed by stage I in 30 % and stage III in 27 % (Fig. 2c).

### 5.2 Assessment of DNA quality and quantity

**Fig. 3:**
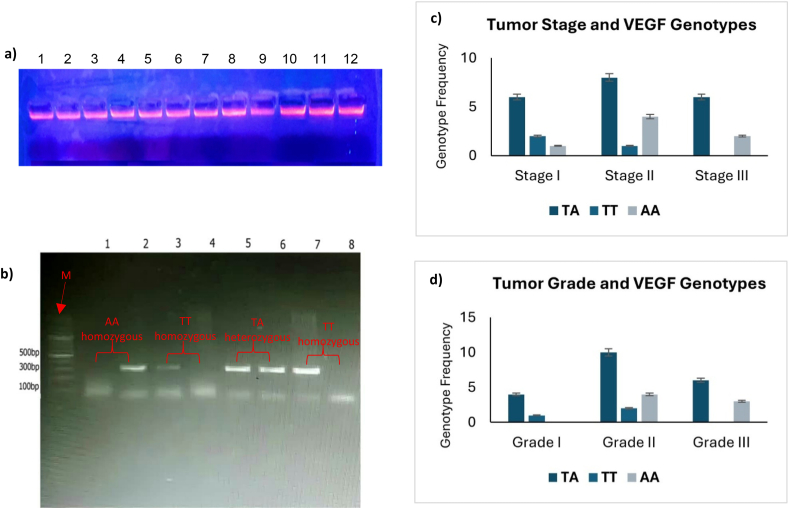
VEGF Genotyping Results. (a Genomic DNA extracted from normal and tumor tissues (lanes 1–12). b) Allele specific PCR gel showing VEGF rs699947 genotypes using wild-type and mutant-specific primers, lane M represents the molecular marker (100 bp DNA Ladder, INTRON). c) Distribution of genotypes across cancer stages. d) Distribution of genotypes across cancer grades. Note: All amplicons are approximately 295–300 bp in size; genotype determination was based on allele-specific primer amplification patterns, not on band size differences.

All tissue samples, 30 normal, 30 tumors, were used for DNA extraction using the guanidine chloride method. DNA quantity and quality were assessed using 1 % agarose gel electrophoresis and a nanodrop device. Most DNA samples exhibited good quality, with concentrations of undiluted samples ranging from 200 to 500 ng/μL (Fig. 3a).

## 6 Genotyping Results

Allele-specific PCR was performed to genotype the VEGF rs699947 polymorphism. All samples showed successful amplification, and genotypes were assigned based on the presence or absence of a ∼295–300 bp band. Each sample was run in two parallel reactions, one with the wild-type-specific primer and one with the mutant-specific primer. Fig. 3b presents a representative gel in which each pair of lanes corresponds to a single patient sample. For example, in lanes 1 and 2, amplification is observed only with the mutant primer (lane 2), indicating a homozygous AA genotype. Lanes 3 and 4 show amplification exclusively with the wild-type primer, representing a TT genotype. Lanes 5 and 6 exhibit bands in both reactions, consistent with a heterozygous TA genotype. Lanes 7 and 8 display amplification only with the wild-type primer, indicating a second TT genotype. These patterns illustrate the genotyping strategy used throughout the study.

The most common genotype among cancer patients was the heterozygous TA genotype, found in 67 % of cases, followed by the homozygous AA genotype (23 %) and the homozygous TT genotype (10 %). In adjacent normal tissue, the TA genotype was also the most common (70 %), followed by the TT genotype (27 %) and the AA genotype (3 %). Statistical analysis revealed no significant difference in genotype frequencies between tumor and normal tissues (P = 0.199) (Table 1). HWE analysis was performed for genotype frequencies in the adjacent normal tissue samples. The genotype distribution significantly deviated from HWE (χ^2^ = 6.96, P = 0.008). This deviation may be attributed to the small sample size, population substructure, or potential technical factors. HWE was not tested in tumor tissues due to the expected genomic instability and clonal expansion inherent to cancer cells. In terms of allele frequency, the T allele was more prevalent in normal adjacent tissues, while the A allele was more common in tumor tissues (57 % vs. 38 %). Although this difference was not statistically significant (Fisher's P = 0.067; Chi-square P = 0.068), the odds ratio of 0.48 (95 % CI: 0.23–0.99) suggests a potential trend toward increased A allele presence in tumor samples (see Table 2).

**Table 1:** Distribution of genotype frequencies between tumor and normal adjacent tissue.

Genotype	Tumor (N = 30)	Normal (N = 30)	OR	95 % CI	Fisher's P-value
TA	20 (67 %)	21 (70 %)	0.86	0.29–2.55	1.000
TT	3 (10 %)	8 (27 %)	0.31	0.07–1.29	0.181
AA	7 (23 %)	1 (3 %)	8.83	1.01–76.96	0.052


Overall Chi-square P-value:	0.199				

∗OR: odd ratio, CI: confidence interval.

**Table 2:** Distribution of allele frequency among tumor and normal tissues.

Study Group	T Allele	A Allele	OR (Tumor vs. Normal)	95 % CI	P (Fisher)	P (Chi-square)
Normal Tissue	37 (62 %)	23 (38 %)	Ref	–	–	–
Tumor Tissue	26 (43 %)	34 (57 %)	0.48	0.23–0.99	0.067	0.068

Distribution of VEGF Genotypes with Histopathology.

Among patients with stage I tumors, the most common genotype was the heterozygous TA genotype (66.6 %), followed by the homozygous wild-type TT genotype (22.2 %) and the homozygous mutant AA genotype (11.1 %). In stage II, the heterozygous TA genotype was again the most frequent (61.5 %), followed by the TT genotype (30.7 %) and the AA genotype (7.6 %). In stage III, the TA genotype was the most common (75 %), followed by the AA genotype (25 %). The distribution of VEGF genotypes by tumor grade showed that the TA genotype was most frequent in grade II tumors (50 %), followed by grade III (30 %) and grade I (20 %). The AA genotype was most common in grade II tumors (57.1 %), followed by grade III (42.8 %). The TT genotype was most frequently observed in grade II tumors (66.6 %), followed by grade I (33.3 %).

## 7 Discussion

VEGF is a potent endothelial cell mitogen, playing a critical role in both normal physiological and tumor angiogenesis. It increases tumor vessel permeability and promotes endothelial cell proliferation, migration, differentiation, and capillary formation, as well as exhibiting proinflammatory actions [24]. The demographic data indicate that middle-aged women, particularly those aged 41–50, are at a higher risk of BC in this study population. This result is consistent with previous studies in Sudan, which also found the highest incidence rate of BC among women aged 40–50 years, with a median age at diagnosis of 45 years [25,26]. These findings suggest that BC occurs at relatively younger ages in this population, with most affected women being under 50 years old. The most plausible explanation for this trend is hormonal changes during the reproductive years. A study in Africa reported that multiparity increases BC risk before age 45 but offers a protective effect after age 45. This explains the age distribution of BC in African women, who tend to have multiple children at a young age [27].

Regarding ethnic origin, most patients in our study were Afro-Asiatic, followed by Nilo-Saharan. A previous study in Sudan identified high-risk areas for BC along the Nile River, Northern, Red Sea, and White Nile states regions, which are predominantly inhabited by Afro-Asiatic tribes, such as the Baniammer, Shaiggy, and Aomara [28]. The predominance of BC cases among the Afro-Asiatic ethnic group suggests potential demographic-specific risk factors such as lifestyle, environment, and inaccessible medical care [29].

Sociocultural factors also play a role, for example, lower awareness about breast health, reliance on traditional medicine, and stigma or fear surrounding cancer can all contribute to later presentation and worse outcomes [28]. Globally, ethnicity and race are recognized as modifiers of BC incidence and outcomes. In the United States, for instance, young black women have a slightly higher incidence of BC under age 35 than their white counterparts and tend to present with more aggressive tumor subtypes and later stages. Similarly, in our context, the predominance of cases in Afro-Asiatic groups might reflect underlying genetic susceptibilities or environmental exposures, but it could also be influenced by disparities in healthcare access. Moreover, it is worth noting that all ethnic groups in Sudan are susceptible to BC; our findings do not imply immunity of other groups but rather reflect the composition of patients who reached diagnostic centres. Nilo-Saharan groups also constituted a significant portion of our cases, consistent with the multiethnic makeup of the region. Nonetheless, the current data highlight the importance of considering ethnic/genetic background and regional factors in BC research.

In terms of family history, 37 % of cases had a familial link to BC, highlighting genetic predisposition, though the majority of cases occurred in patients without such a history. A previous study in the Sudanese population found that only 15 % of patients had a family history of BC [26]. This may suggest that genetic mutations from aging and other factors, rather than inherited mutations, contribute to BC, highlighting its multifactorial nature [30]. Globally, it is estimated that the majority of BC cases are sporadic, while a smaller fraction involves a strong inherited genetic component (such as BRCA1/2 mutations or other hereditary syndromes) [31]. [32]. While hereditary risk remains an important factor, our data suggest that non-genetic factors predominate in this cohort. BC research has been increasingly focused on inherited risk factors such as family history and genetic mutations, especially in housekeeping genes [33].

IDC is the predominant type observed in this study, similar to other studies conducted in Morocco and Sudan, which showed the predominance of IDC in histopathological data [18,34]. Similarly, research in Sub-Saharan Africa reported that IDC accounted for 75 % of total BC cases [29]. A study in the Jordanian Arab population showed that incidence median age was 51, with 30 % of the patients with a family history, and 82 % had IDC [35]. IDC is the most common BC histology globally (usually 70–80 % of cases in most registries), although the exact percentage can vary slightly by region [36,37].

Regarding tumor stage in this study, stage II was the most commonly observed tumor stage among female patients. Similarly, a previous study found that stage II BC was the most frequent among Nigerian women, representing 48.57 % of the cases [38]. In the Jordanian Arab population, 90 % of cases were diagnosed at a lower tumor stage [39]. Earlier studies in Sudan have shown that stage III is the most common, likely due to limited awareness, challenges in accessing healthcare services, and the absence of a cancer screening program [4,28,34]. However, there has been a slight improvement, possibly due to screening programs using local volunteers, leading to increased detection of BC in asymptomatic women in low-income rural areas. Over the past decade, multiple BC awareness campaigns and screening initiatives (sometimes driven by local NGOs and volunteer health workers) have been implemented in Sudan. These efforts, ranging from public education about breast self-exam to mobile clinics in rural areas, likely contributed to more women seeking evaluation of breast lumps earlier than before. Indeed, there have been community-based screening activities (often involving local female volunteers) in some low-income and rural areas, which have helped in detecting asymptomatic or smaller tumors that otherwise might have gone unnoticed until becoming advanced. While Sudan still lacks a formal nationwide mammography screening program, these grassroots efforts and improved healthcare outreach could explain the slight stage shift.

A key aim of our study was to examine the distribution of the VEGF-2578 C > A polymorphism (rs699947) in BC tissue and adjacent normal tissue, and to test for any association with BC risk. Our study revealed that in tumor tissues, the most common genotype was TA (heterozygous), followed by AA (mutant) and TT (wild). In adjacent normal tissue, the TA (heterozygous) genotype was also the most frequent, followed by TT (wild) and AA (mutant). The mutant allele (A) was more prevalent in tumor tissue, whereas the wild-type allele (T) was more commonly found in the adjacent normal tissue from the same patient. Our data analysis showed no significant association between different VEGF genotypes and BC, which contrasts with some literature. For instance, Thammineni et al. reported a significant association between VEGF gene polymorphism and increased BC risk in North-West Indians [26]. Another study in the Iraqi population showed that the A allele is more frequent in the malignant group compared to the control group and is associated with an increased risk of BC [17]. Another case-control study in Saudi females showed that the A allele also increased the risk for BC [14]. Additionally, a study in the Iranian population showed that the AA variant increases the risk of BC and significantly decreases the risk of developing BC negative for human epidermal growth factor receptor 2 (HER2), which promotes cancer cell growth [16]. Moreover, the frequency of the A allele in our study was 57 % in BC tissue and 38 % in normal tissue, which was higher compared to other studies in which the prevalence was 28 % in the USA, 28 % in Poland, 26 % in Sweden, 25 % in England, and 4 % in Morocco [14]. This ethnic variation in allele frequency is crucial because a polymorphism's effect on disease risk can depend on how prevalent that polymorphism is and on the genetic context [40]. In our study, even though the A allele was relatively common, we did not find a significant risk association. It's possible that in this population, other genetic or environmental factors overshadow the influence of this single SNP.

On the other hand, consistent with our results, Rahoui et al. showed that the A allele was associated with a reduced risk of developing BC [18]. Another large case-control study in the United States revealed that VEGF was not significantly associated with BC in pre- and postmenopausal women [25]. Similarly, a meta-analysis by Chen et al. found no overall association between the −2578C > A variant and breast or bladder cancer risk in combined populations [41]. Another meta-analysis did not find a significant impact of this SNP on BC susceptibility [42]. These suggest that, on a broad scale, this polymorphism alone may not be a strong universal risk factor.

The nature of BC carcinogenesis is complex, and there is no clear cause for the discrepancies in different studies. However, ethnic, genetic, and environmental factors can interact in various ways to influence the risk of BC in different populations.

## 8 Study limitation

Large-scale studies in the Sudanese population are essential for definitive confirmation of our findings. One key limitation of this study is the relatively small sample size, which may reduce the statistical power to detect modest associations between VEGF polymorphisms and BC risk. This limitation underscores the need for larger, well-powered studies to validate our findings and better understand the genetic basis of BC in the Sudanese population. Our study can lay the groundwork for future research with larger sample sizes to verify and strengthen our findings.

## 9 Conclusion

Early diagnosis of BC is essential for reducing morbidity and mortality. The actual incidence of BC in Sudan remains unknown due to the lack of a national cancer registry. Conflicting results have emerged globally regarding the association between the VEGF (rs699947) gene polymorphism and BC. This case-control study is the first to investigate this gene polymorphism among Sudanese women, aiming to enhance understanding of BC development. Our demographic data indicated that BC tends to occur at younger ages (41–50 years), with predominance among the Afro-Asiatic ethnic group, which suggests demographic-specific risk factors. IDC was the most common type, and stage II was the most frequently diagnosed tumor stage, reflecting the need to improve early detection efforts. Despite the higher prevalence of the A allele in tumor tissues, no significant association was found between VEGF genotypes and BC risk, suggesting that ethnic, genetic, and environmental factors contribute to BC complex nature in this population. While this study provides initial insights into VEGF polymorphisms and BC risk among Sudanese women, the small sample size remains a major limitation, and future investigations with larger cohorts are essential to confirm and extend these findings.

## CRediT authorship contribution statement

**Hala Osman:** Writing – original draft, Software, Methodology, Investigation, Conceptualization. **Mozan Hassan:** Writing – review & editing, Visualization. **Mohamed Alfaki:** Validation, Software, Data curation. **Ghada Haj-Ali:** Writing – review & editing, Validation, Supervision, Conceptualization.

## Declaration of competing interest

The authors declare that they have no known competing financial interests or personal relationships that could have appeared to influence the work reported in this paper.

## Data Availability

Data will be made available on request.
